# Editorial: From sunlight to plant health: decoding metabolic responses

**DOI:** 10.3389/fpls.2025.1741911

**Published:** 2025-12-01

**Authors:** Carmen Arena, Luigi Gennaro Izzo, Marina López-Pozo

**Affiliations:** 1 Department of Biology, University of Naples Federico II, Naples, Italy; 2 Department of Agricultural Sciences, University of Naples Federico II, Portici, Italy; 3 Department of Ecology & Evolutionary Biology, University of Colorado, Boulder, CO, United States

**Keywords:** light, secondary metabolism, primary metabolism, photosynthesis, pigment synthesis, antioxidants

Light is one of the most fundamental and dynamic environmental factors driving the development of plant form and function. Beyond its role as the primary energy source for photosynthesis, light functions as a master regulator of development, physiology, and metabolic networks. Plants have evolved sophisticated mechanisms to perceive and respond to changes in light intensity, spectrum, and duration, allowing them to fine-tune both primary and secondary metabolic pathways. Therefore, understanding how light modulates plant metabolism is central, not only to plant physiology but also to ecology and global sustainability. With this Research Topic, From Sunlight to Plant Health: Decoding Metabolic Responses, we aimed to gather multidisciplinary perspectives that bridge the molecular, physiological, and ecological dimensions of how plants harness and adapt to light.

The articles collected in this Research Topic illustrate how light quality and quantity act as powerful modulators of plant growth, shaping their metabolism through complex networks of biochemical, transcriptional, and hormonal pathways. These contributions underscore the necessity of integrative approaches spanning from molecular signaling to whole-plant performance and ecosystem-level implications for deciphering light-driven metabolic responses.

A compelling example of this multi-scale inquiry is presented in the original research by Menicucci et al., who investigated the impact of specific LED light spectra on two cultivars of red-leaf chicory (*Cichorium intybus*), which is rich in vitamins, minerals, and nutraceutical compounds. Their findings reveal striking differences between the cultivars and the various light treatments. The study shows that blue light consistently enhanced the content of photosynthetic pigments and boosted the accumulation of antioxidant polyphenols, such as quercetin derivatives, compared to red and white LEDs. In contrast, red light not only reduced Photosystem II efficiency but also shifted the polyphenolic profile toward a predominance of kaempferol derivatives. These results underscore that selecting specific light spectra can effectively modulate the production of high-value nutraceutical metabolites in a species-specific manner. Importantly, this study emphasizes that plant metabolic plasticity depends not only on light conditions but is also tightly linked to genetic background, highlighting the need for species- and cultivar-specific protocols when manipulating light environments to enhance plant performance.

Complementing these experimental insights, the review by Wu et al. provided a comprehensive view of the role of light in regulating plant growth, development, and sugar metabolism. This work summarizes how light quality, intensity, and photoperiod affect sugar production, partitioning, and signaling, including how light signals drive the activity of key enzymes involved in sucrose synthesis and degradation (e.g., SUS, SPS, and INV) in several species (melon, sweet cherry, and banana). These light-controlled signals play a key role in carbon partitioning, influence mineral uptake, and orchestrate resource allocation between source and sink tissues, representing a fundamental process underlying plant yield and quality. Their review bridges the gap between photosynthetic activity and carbohydrate metabolism, illustrating that light influences not only energy capture but also the allocation and utilization of carbon resources. This integrative overview emphasizes that the effects of light on primary metabolism are both direct, through photosynthetic efficiency, and indirect, via transcriptional and hormonal control of metabolic fluxes.

At a more mechanistic and biophysical level, Murakami et al. addressed a question that sits at the intersection of photoprotection, energy dissipation, and ecosystem/global climate relevance: how much heat is produced by non-photochemical quenching (NPQ)? This theoretical and modeling-based study quantified the magnitude of heat release associated with NPQ under natural sunlight conditions. Although the direct warming effect at the leaf level was modest, their work reveals that NPQ-induced thermal radiation from vegetation can represent a measurable component of Earth’s energy balance. Interestingly, the authors found that a small, yet non-negligible fraction (up to 0.55%) of the total thermal radiation emitted from the Earth’s surface originates from NPQ, highlighting it not only as a photoprotective mechanism in plants but also as a minor, yet noteworthy, contributor to biosphere-atmosphere interactions. This study broadens our understanding of NPQ, framing it not merely as a physiological defense mechanism but as a process with potential ecological and even atmospheric implications, thereby demonstrating that photosynthetic regulation links micro- and macroscale processes in unexpected ways.

Finally, the opinion article by Aguirre-Böttger and Zolla adopted a broader evolutionary and applied perspective, focusing on *Solanaceae* biodiversity as a key resource for sustainable food and cosmetic production. Their contribution contextualizes photosynthetic efficiency and metabolic diversity within the broader challenges of climate change and food security. Emphasizing the need for sustainable food and cosmetic industries, the authors proposed that wild relatives and underutilized species display crucial genes for enhancing photosynthetic performance (e.g., related to PSII stability, RuBisCO kinetics, and electron transport) and the production of secondary metabolites, such as lycopene, under climate change-induced stress conditions. This perspective powerfully links the conservation of genetic diversity with the practical goal of engineering crops that are both more productive and resilient, identifying light-use efficiency as a key target.

In conclusion, the contributions gathered in this Research Topic reflect the wide diversity of approaches currently used to study light-driven metabolism, from detailed metabolic profiling and gene expression analysis to theoretical climate modeling, biodiversity-based strategies, and applied technologies. Despite their methodological diversity, these studies share a common thread: light functions as an integrative signal linking molecular, physiological, and ecological scales within the plant kingdom.

As Topic Editors, we believe that the path forward lies in strengthening the connections across these scales. Understanding how light quality and quantity shape plant metabolic networks requires not only the study of individual pathways but also an assessment of their interconnections, from the absorption of photons by pigments to ecosystem-level productivity. Emerging technologies in photobiology, multi-omics, and computational modeling are currently enabling a more holistic exploration of these relationships. Furthermore, integrating this knowledge into dynamic models that predict plant behavior in fluctuating natural environments will be crucial. This comprehensive understanding will shed new light on strategies to improve crop productivity, enhance the nutritional value of plants, and better predict the role of vegetation in a changing climate.

